# Utilizing Gold Nanoparticle Probes to Visually Detect DNA Methylation

**DOI:** 10.1186/s11671-016-1487-5

**Published:** 2016-06-21

**Authors:** Kui Chen, Mingyi Zhang, Ya-Nan Chang, Lin Xia, Weihong Gu, Yanxia Qin, Juan Li, Suxia Cui, Gengmei Xing

**Affiliations:** CAS Key Laboratory for Biomedical Effects of Nanomaterials and Nanosafety, Institute of High Energy Physics, Chinese Academy of Science (CAS), Beijing, 100049 China; School of Life Sciences, Capital Normal University, Beijing, 100048 China

**Keywords:** DNA methylation, Gold nanoparticles probe, Surface plasmon resonance, Visual detection, Semi-quantitative assay

## Abstract

The surface plasmon resonance (SPR) effect endows gold nanoparticles (GNPs) with the ability to visualize biomolecules. In the present study, we designed and constructed a GNP probe to allow the semi-quantitative analysis of methylated tumor suppressor genes in cultured cells. To construct the probe, the GNP surfaces were coated with single-stranded DNA (ssDNA) by forming Au–S bonds. The ssDNA contains a thiolated 5′-end, a regulatory domain of 12 adenine nucleotides, and a functional domain with absolute pairing with methylated *p16* sequence (Met-*p16*). The probe, paired with Met-*p16*, clearly changed the color of aggregating GNPs probe in 5 mol/L NaCl solution. Utilizing the probe, *p16* gene methylation in HCT116 cells was semi-quantified. Further, the methylation of *E-cadherin*, *p15*, and *p16* gene in Caco2, HepG2, and HCT116 cell lines were detected by the corresponding probes, constructed with three domains. This simple and cost-effective method was useful for the diagnosis of DNA methylation-related diseases.

**Electronic supplementary material:**

The online version of this article (doi:10.1186/s11671-016-1487-5) contains supplementary material, which is available to authorized users.

## Background

DNA methylation is an important regulator of gene expression, and its role in tumorigenesis has been a central topic in the last few decades [1]. Many studies have indicated that hypermethylated CpG islands in tumor suppressor gene promoter sites can increase chromosome coiling and gene silencing, and this process occurs prior to malignant cell growth [2, 3]. As site-specific methylation occurs early and can be detected even in body fluid, it is regarded as a potential biomarker for early tumor detection and determining prognosis [4–6].

To date, numerous techniques have been developed for DNA methylation detection [7, 8]. Due to the particular physicochemical property of nanoscale materials, nano-based DNA methylation detection has emerged as an important option. The specific optical properties of nanomaterials change the very foundation of traditional DNA methylation sensing [9–11]. Among the abundant types of nanomaterials, gold nanoparticles (GNPs) are most extensively applied due to their unique chemical and physical properties that are strongly dependent on their size, shape, and degree of aggregation [12]. Colorimetric assays based on GNP surface plasmon resonance are more applicable as clinical markers because they only require a UV/vis spectrometer. Most studies have concentrated on indirect methods to detect DNA methyltransferases or DNA methylases based on GNPs [13–15]. Zeng’s group used antibody-conjugated magnetic microspheres to capture methylated DNA [16]. After their release from the microsphere by heat denaturation, methylated DNA was added to unmodified GNPs to prevent GNPs from aggregating in the salt solution, whereas the non-methylated group cannot be captured, and no DNA was released into the GNP solution. One limitation of this method is the need of an antibody to recognize the methylated DNA sequence. In this work, we tried to construct a highly sensitive single-stranded DNA (ssDNA)-GNP probe to detect DNA methylation in cultured cells.

**Fig. 1:**
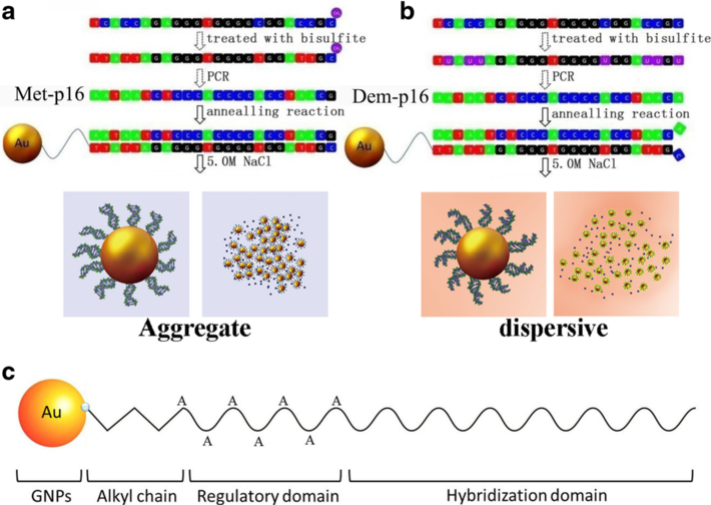
The principle of DNA methylation detection by GNPs based on the SPR effect (**a**, **b**) and a schematic illustration of the composition of ssDNA-GNP probe (**c**)

*p16* are tumor suppressor genes and their transcription activities can be inhibited by hypermethylation in the promoter site. Therefore, the small CpG region in the promoter site was selected as the target to test the probe and achieve semi-quantitative detection of DNA methylation. We designed an ssDNA-GNP probe to target and visually detect the CpG region of the tumor suppressor genes by introducing a colorimetric method to modify an existing bisulfite-based method that measures CpG region methylation. After the sequence was treated with bisulfate, the 5′-ends were C-CH_3_ and U in the methylated and non-methylated DNA sequences, respectively. Their polymerase chain reaction (PCR) products (Met-*p16* and Dem-*p16*, respectively) were used as standard sequences in the following experiment to test the probe and calculate a standard curve for semi-quantitative detection of intracellular DNA methylation (Table 1). We applied the aggregation principle reported by Sato et al. [17] and Liu and Lu [18] to construct an ssDNA-GNP probe. The probe contained a sequence that absolutely paired with Met-*p16* but mismatched Dem-*p16* at the terminal base. This assay consisted of two steps (Fig. 1a, b): (a) the target sequences (Met-*p16* and Dem-*p16*) were added to the ssDNA-GNP probe solution and incubated on ice for 1 h, and then Tris-acetate buffer containing NaCl was used to regulate the salt concentration of the final colloidal solution of GNPs and (b) GNP aggregation behavior was investigated by observing the solution color and monitoring the UV-vis absorption spectrum. We then drew a standard curve of correlation between the Met-*p16* content and the UV-vis spectra of *A*_620 nm_/*A*_520 nm_ in the probe solution. Following the process, *p16*, *E-cadherin*, and *p15* ssDNA-GNP probe with three domains were constructed and utilized to assay the methylation of these tumor suppressor genes in three cancer cell lines. The results show that the ssDNA-GNP probe can semi-quantitatively detect DNA methylation. Moreover, the ssDNA-GNP probe can be designed to pair with various oligonucleotides for the specific detection of different (and even multiple) methylated target genes.

**Table 1:** Sequences of oligonucleotides used in the assay

Met-p16	cgc cac cac cct cca acc t
Dem-p16	tgc cac cac cct cca acc t
Probe1	SH-(CH_2_)_3_-aaa aaa aaa aaa tta ttt agg ttg gag ggt ggt ggc g
Probe2	SH-(CH_2_)_3_-aaa aaa tta ttt agg ttg gag ggt ggt ggc g
Probe3	SH-(CH_2_)_3_-tta ttt agg ttg gag ggt ggt ggc g
MPs p16	gtt ttt tag aat gtt ggg att ata ga
MPa p16	ctc aaa aaa cta aaa caa aaa aat c
NPs	ttg tta ttt agg ttg gag ggt ggt
PC p16	tta ttt agg ttg gag ggt ggt ggc gcg att tcg gtt tat tgt aat ttt tgt ttt tcg gg
NC p16	tta ttt agg ttg gag ggt ggt ggt gtg att tcg gtt tat tgt aat ttt tgt ttt tcg gg
Probe E-cad	SH-(CH_2_)_3_-aaa aaa tta ggt tag agg gtt atc g
MPs E-cad	ttt agt aat ttt agg tta gag ggt tat
MPa E-cad	aaa ctc aca aat act tta caa ttc c
PC E-cad	taa ttt tag gtt aga ggg tta tcg cgt tta tgc gag gtc ggg tgg gcg ggt cgt tag
NC E-cad	taa ttt tag gtt aga ggg tta ttg tgt tta tgc gag gtc ggg tgg gcg ggt cgt tag
Probe p15	SH-(CH_2_)_3_-aaa aaa gat tat tcg ggt cgt tgc g
MPs p15	agg aga ata agg gta tgt tta gtg g
MPa p15	ccc taa aac ccc aac tac cta aat
PC p15	acg gtg gat tat tcg ggt cgt tgc gcg ttt ggg ggt tgc gga atg cgc
NC p15	acg gtg gat tat tcg ggt cgt tgt gtg ttt ggg ggt tgc gga atg cgc

Note: Met-p16 and Dem-p16 correspond to a partial methylated and demethylated DNA sequence of p16, respectively. A partial *E-cadherin*, *p15*, and *p16* sequences were amplified with MPs E-cad and MPa E-cad, MPs p15 and MPa p15, MPs p16 and MPa p16 primers, respectively, that were identified from Methprimer. NPs and P16MPa primers were used to perform nested PCR. PC and NC sequences were all dsDNA. The special cutting site for *Bst*UI could be found in the PC group but not in the NC group. Thus, PC E-cad, NC E-cad, PC p15, NC p15, PC p16, and NC p16 sequences were used as positive and negative controls during the DNA methylation detection of E-cadherin, p15 and p16, respectively

## Methods

### Reagents and Chemicals

Chloroauric acid (HAuCl_4_·4H_2_O) and trisodium citrate were obtained from Sinopharm Chemical Reagent Beijing Company Limited (Beijing, China). Tris (2-carboxyethyl) phosphine (TCEP) was obtained from Sigma-Aldrich (St. Louis, MO, USA). All oligonucleotides designed in this study were synthesized by Sangon Biotech Company Limited (Shanghai, China). The sequences of oligonucleotides are presented in Table 1. Other reagents were all of analytical grade.

### GNP Synthesis and ssDNA-GNP Construction

GNPs (~13 nm) were synthesized using a citrate reduction method [19]. Briefly, 200 mL of 1 % (wt) HAuCl_4_ was brought to boil in a 250-mL flask. Next, 1.2 mL 5 % (wt) sodium citrate was added under rapid stirring with a magnetic stir bar. After the solution color turned from purple to red, the boiling and stirring were allowed to continue for 5 min. When the solution was cooled to room temperature, GNP aliquots were centrifuged at 7000*g* and resuspended in ddH_2_O. The GNP concentration was determined by UV-vis spectrophotometer [20].

The ssDNA-GNP probes were constructed as described by Liu and Lu [18]. First, thiolated ssDNA was activated by freshly prepared TCEP (10 mM in acetate buffer, pH 5.2), and 30 μL ssDNA (100 μM) was added into 1 mL GNP solution (57 nM). The reaction solution was incubated at room temperature for at least 16 h with gentle shaking. Then, Tris-acetate buffer (500 mM, pH 8.2) and 100 μL NaCl (1 M) was added to the tubes dropwise with gentle shaking until the concentration of Tris-acetate reached 5 mM. The synthesized ssDNA-GNP probes were stored in the dark for 24 h, and then collected by centrifugation at 8000*g* at room temperature for 15 min.

### GNPs and ssDNA-GNP Characterization

Scanning electron microscopy (SEM) was performed on a Hitachi S-4800 analyzer (Hitachi, Tokyo, Japan), and particle size distributions were evaluated using a NicompTM 380 DLS particle size analyzer (Nicomp, Santa Barbara, CA, USA). Agarose gel electrophoresis assays were performed to assess the dispersal of GNP and ssDNA-GNP probes [21]. After mixing 5 μL GNP or ssDNA-GNP samples with 1 μL glycerol and 4 μL ultrapure water, the mixtures were loaded on a 1 % agarose gel buffered with 1 × TAE (Tris-acetate-EDTA, pH 8.3). Agarose gel electrophoresis was run in 1× TAE buffer at a constant 120-V voltage for 20 min. The electrophoresis results were imaged with a digital camera (OLYMPUS E-520, Tokyo, Japan).

To determine the number of ssDNA per GNP, DTT was added into the probe solution to release the immobilized ssDNA. The probe solution was incubated at room temperature for 10 min, and the GNPs rapidly aggregated. After removing the GNPs by centrifugation at 14,000*g* for 5 min, the released ssDNA in the supernatant was quantified using QuantiFluor ssDNA system (Promega, Madison, WI, USA).

To compare the stability of nude GNPs and the ssDNA-GNP probe, we prepared Tris-acetate buffers containing NaCl concentrations from 0.1 to 2.5 M. Next, 10 μL GNPs or probes (200 nM) were added to 300 μL Tris-acetate buffers and incubated for 10 min at room temperature. The absorbance spectra of GNP solution and probe solution were recorded from 400 to 800 nm on a UV-vis spectrometer (Persee General, Beijing, China).

### Testing of the Probe and Drawing of Standard Curve

Two ssDNA samples (Met-*p16* and Dem-*p16*, Table 1) were applied as substrates and diluted to 100 μM. Each DNA sample (2 μL) was mixed with Tris-acetate buffer containing 0.1 M NaCl and 200 nM probes to a total volume of 1 mL. The solutions were firstly incubated on ice for 1 h and then incubated at room temperature for 10 min. After removing residual DNA samples by centrifugation at 8000*g* (20 min at 4 °C), the precipitation was resuspended in Tris-acetate buffer containing NaCl (concentration range from 0.1 to 5.0 M) and incubated at room temperature for 5 min. The absorbance spectra of the products were recorded from 400 to 800 nm on a UV-vis spectrometer. We then prepared sample solutions containing different ratios of Met-*p16* (0–100 μM) and Dem-*p16* (0–100 μM). The final products were resuspended in Tris-acetate buffer containing 5.0 M NaCl. Other protocols were followed as mentioned above. Based on these results, we drew a standard curve of correlation between Met-*p16* level and the probe solution’s UV-vis absorption spectrum at *A*_620 nm_/*A*_520 nm_.

### The ssDNA-GNP Probe of *E-cadherin* and *p15* Gene

To detect DNA methylation of *E-cadherin* and *p15* gene, the ssDNA-GNP probe was constructed. Based on the result of the probe of *p16* gene, the ssDNA contain three domains: a hybridization sequence for targeting the promoter site of *E-cadherin* and *p15* suppressor gene; a mercapto group at the 5′-end for linking ssDNA onto GNPs with an Au-S bond; and a regulatory region of 12A to avoid the unspecific adsorption between Au and those elements. Using freshly prepared TCEP (10 mM in acetate buffer, pH 5.2) to activate thiolated ssDNA was the first step. Then, 30 μL ssDNA (100 μM) was added into 1 mL GNP solution (57 nM), and the mixed solution was incubated at 37 °C for 16 h under gentle shaking. Next, Tris-acetate buffer (500 mM, pH 8.2) and NaCl (1 M) were dropwise added into the reaction tubes to regulate the concentration of Tris-acetate to 5 mM. The synthesized ssDNA-GNP probes were stored in the dark for 24 h, and then collected by centrifugation at 8000*g* at room temperature for 15 min.

### Genomic DNA Extraction and Bisulfite Treatment

Genomic DNA from CaCo2 (human colon cancer cell line), HepG2 (human hepatocellular carcinoma cell line), and HCT116 cells (human colon cancer cell line, China Infrastructure of Cell Line Resources; Chinese Academy of Medical Sciences, Beijing, China) were extracted using a genomic DNA extraction kit according to the manufacturer (TIANGEN, Beijing, China). Bisulfite treatment DNA was accomplished by an EZ DNA methylation kit according to the manufacturer (Zymo, Irvine, CA, USA).

### PCR Amplification and Digestion with BstUI

*E-cadherin*, *p15*, and *p16* were amplified with bisulfite primers (Table 1) from bisulfite treatment DNA. PCR conditions were as follows: 95 °C for 5 min; then 35 cycles of 95 °C for 30 s, 55 °C for 30 s, 72 °C for 30 s; and finally, 5 min at 72 °C. To obtain products for detection (*p16*), a second round of PCR was performed with a pair of nested primers (Table 1) under the same conditions.

The PCR product was digested by *Bst*UI (New England Biolabs, Ipswich, MA, USA) overnight according to the manufacturer’s protocol. To facilitate comparison, negative and positive controls (NC and PC seq, respectively; Table. 1) were also digested. The NC seq could not be digested by *Bst*UI, but the PC seq with a special site (CGCG) could be digested.

### DNA Methylation Detection in Cultured Cell Lines

The mixtures produced with the above-described treatment were denatured at 95 °C, and then were added to the ssDNA-GNP probe solution. The reaction was performed according to the program, which was published in the probe testing system. After 5 min, the products’ absorption spectra from 400 to 800 nm were measured, and images of the solution were captured.

### Statistics

All results were calculated as mean ± SD unless otherwise specified. Data are reported as biological replicates except for hydrodynamic size analysis, where technical replicates from a representative experiment were used. Data were analyzed with IBM SPSS Statistics (Chicago, US) using one-way analysis at *P* < 0.05.

## Results and Discussion

### Linking ssDNA onto GNPs

**Fig. 2:**
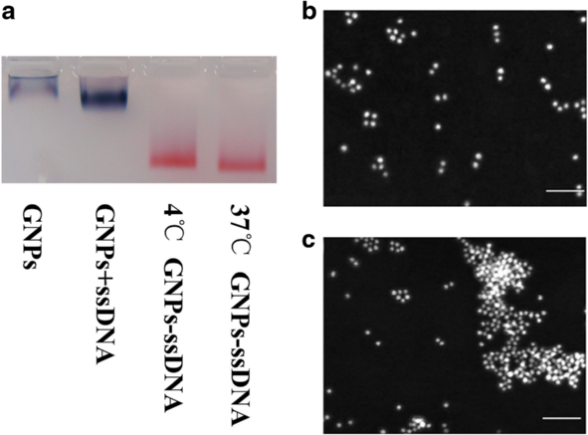
Influence of temperature on ssDNA-GNP stability. **a** Agarose gel images of different ssDNA-GNP samples. **b**, **c** SEM images of ssDNA-GNPs synthesized at 37 and 4 °C, *scale bar* = 100 μm

Functionalizing 13-nm GNPs with ssDNA was a crucial step in constructing the probe used in our experiment. The specially designed oligonucleotides included a hybridization domain that targeted a sequence in the promoter site of the *p16* tumor suppressor gene and a mercapto group at the 5′-end. Linking ssDNA onto GNPs with an Au–S bond was carried out at 4 or 37 °C. Then, agarose electrophoresis and SEM were used to evaluate and compare the stabilities of these ssDNA-GNPs in 0.1 mol/L NaCl solution (Fig. 2). The bands of samples present different colors from red to blue and various migration distances in agarose gel electrophoresis. The blue was induced by particle aggregation and was associated with short migration distance (Fig. 2a). Functionalized GNPs with ssDNA were red in the gel, indicating that linking ssDNA onto the GNPs improve their stability. The ssDNA-GNPs synthesized at 37 °C (Fig. 2b) performed more monodispersity and more stability than ssDNA-GNPs synthesized at 4 °C (Fig. 2c). We also evaluated the stability of ssDNA-GNPs generated at 50 °C. Both agarose electrophoresis and SEM illustrated that these ssDNA-GNPs were less stable and had poorer monodispersal (Additional file 1: Figure S1). Compared with other particles, the ssDNA-GNPs synthesized at 37 °C had the best stability, so this temperature was used to construct probes for the remaining experiments.

### Construction and Characterization of the ssDNA-GNP Probe

The GNP probe structure is shown in Fig. 1c. Besides the mercapto group and hybridization domain, the specially designed ssDNA in the probe included a regulatory domain. We designed three probes with different numbers of adenine nucleotide (A) in the regulatory domains (12, 6, and 0 in probes 1, 2, and 3, respectively) and visually evaluated their stability in 0.1 and 5.0 mol/L NaCl solution. The regulatory domain plays a key role in GNP stability by affecting the double electrode layer [12]. ssDNA with different regulatory domains were linked onto the GNPs at the optimal temperature (37 °C) by Au–S bonds. These ssDNA-GNPs were added into saline solution to observe their stability. The colors of probes 1 and 2 were red in 0.1 mol/L NaCl, and there was no broadening in their UV/vis absorption peaks (Additional file 1: Figure S2a, b). The aggregation of probe 1 was induced by the hybridization of targeted DNA and appeared blue in the 5.0 mol/L NaCl solution. Probe 1 was much more sensitive than probe 2, because the latter was dispersible, and the solution was still red in the same condition. Therefore, we hypothesize that the repeated As in the regulatory domain influenced probe stability and further impacted the ability of the probe to assay the targeted sequences in the salt solution. Besides, phosphate, oxygen, and nitrogen in deoxyribonucleic can also form coordinate bonds with Au [22, 23]. The regulatory region may assist to avoid the unspecific adsorption between Au and those elements. Thus, we determined that the optimal regulatory domain was 12A in probe 1.

**Fig. 3:**
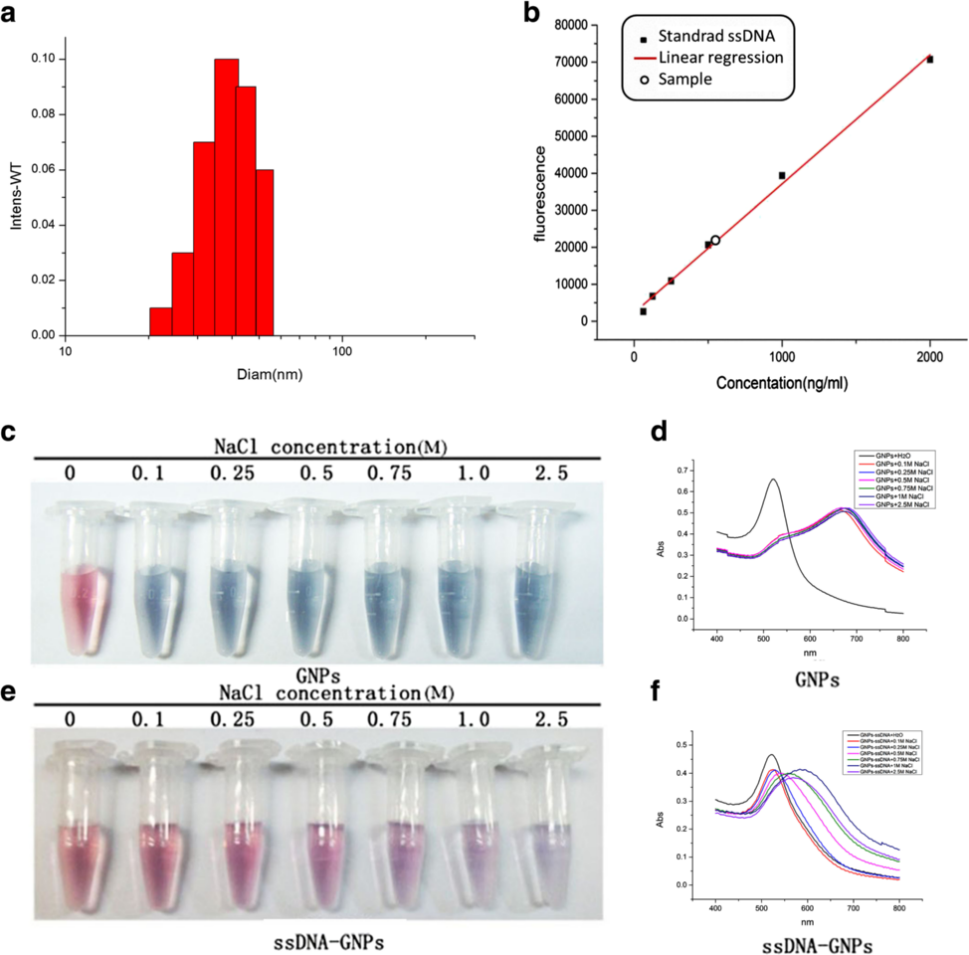
Characteristics of ssDNA-GNP probes. **a** The hydrate particle size was determined by DLS. **b** Estimation of the ratio of immobilized ssDNA on pre-GNPs. The stability of nude GNPs (**c**) and ssDNA-GNPs (**e**) in various concentrations of salt solution. Changes in UV-spectrum (**d**, **f**)

The probe was also characterized by dynamic light scattering (DLS). The results showed that the probe had excellent monodispersity and uniform particle structure. The hydrated particle size of the probes was 38.3 ± 4.4 nm (Fig. 3a). The amount of ssDNA coupled to the GNPs was also determined by UV analysis [24]. The molar ratio of GNPs and ssDNA was 1:18 ± 1 in the probe under the optimum synthesis condition (Fig. 3b).

The accurate stability and monodispersity of the ssDNA-GNP probes was further evaluated for their usefulness in detecting DNA methylation. When the probe was added into different NaCl solution concentrations (0.1, 0.25, and 0.5 mol/L), the solution color remained red. A gradual change in solution color from red to purple and finally to blue was observed with increased NaCl concentration from 0.75 to 2.5 mol/L (Fig. 3c–f). In contrast, the color of the nude GNPs instantaneously changed from red to blue even at a low NaCl concentration (0.1 mol/L). In addition, the aggregation of the nude GNPs induced a broadening absorption peak and a 144-nm red shift in the UV/vis spectrum. As a result, the probe was more stable in the salt solution. A previous study showed that the ssDNA-GNP probe with 159 DNA sequence on one nanoparticle was stable but not sufficient sensitive [17]. Therefore, it was our goal to construct a probe with an appropriate balance of stability and sensitivity.

### Standard Curve to Assay DNA Methylation

**Fig. 4:**
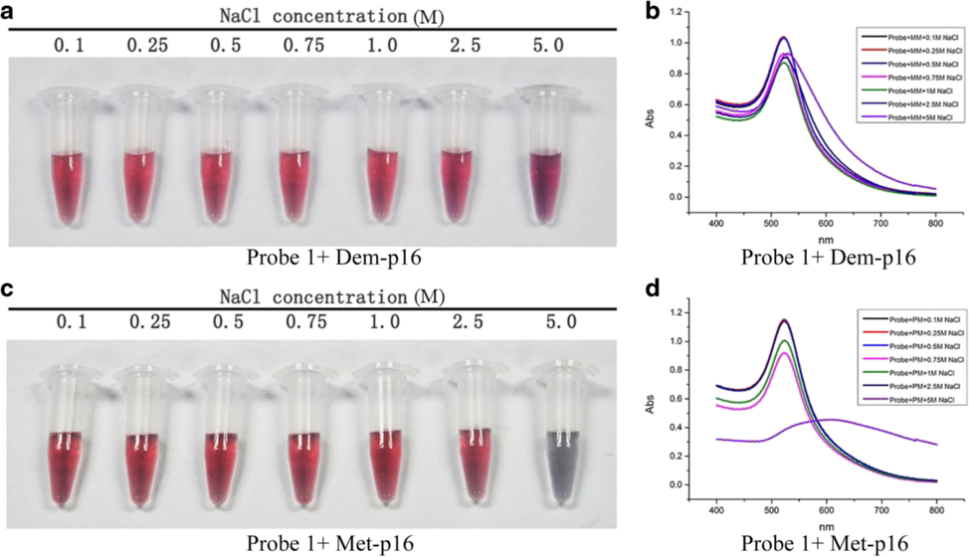
Visual determination of DNA methylation in the mimic system. Visual detection of Dem-*p16* (**a**) and Met-*p16* (**c**) in various salt concentrations. Changes in UV-spectrum (**b**, **d**)

We utilized *p16* probe 1 to detect methylated DNA. The probes manifested different colors after incubation and annealing with Met-*p16* or Dem-*p16* because the distinct structures of the two dsDNA-GNPs induced variable particle aggregation in salt solution after ssDNA pairing (Fig. 4 and Additional file 1: Figure S3). The dsDNA-GNPs from the pairing probe with Dem-*p16* were stable in the NaCl solution from 0.1 to 5.0 mol/L (Fig. 4a and Additional file 1: Figure S3a), and the absorbance spectra showed litter change in both groups (Fig. 4b and Additional file 1: Figure S3b). In contrast, dsDNA-GNPs paired with Met-*p16* aggregated in the 5.0 mol/L NaCl solution (Fig. 4c and Additional file 1: Figure S3c), with the color changing from red to blue. The obvious peak broadening and red shift (from 522 to 575 nm) were noted in the UV/vis absorbance spectra (Fig. 4d). However, probe 2 did not induce a change in solution color or a pronounced red shift of the absorbance spectra peak (from 522 to 544 nm) when it formed dsDNA-GNPs with Met-*p16* in the salt solution (Additional file 1: Figure S3d). The final ssDNA-GNP probe architecture contained a thiolated 5′-end, a regulatory domain of 12A nucleotides, and a functional domain with absolutely pairing match with Met-*p16*.

Though the interpretation of the phenomenon was not clear, we hypothesized that a single-base mismatch at the outermost position of the ssDNA-GNP probes may induce a repulsive interaction [17]. The repulsive interaction was amplified by the GNPs’ huge specific surface area. Ultimately, the variable stability between these two dsDNA-GNPs was large enough to be observed by the naked eye.

**Fig 5:**
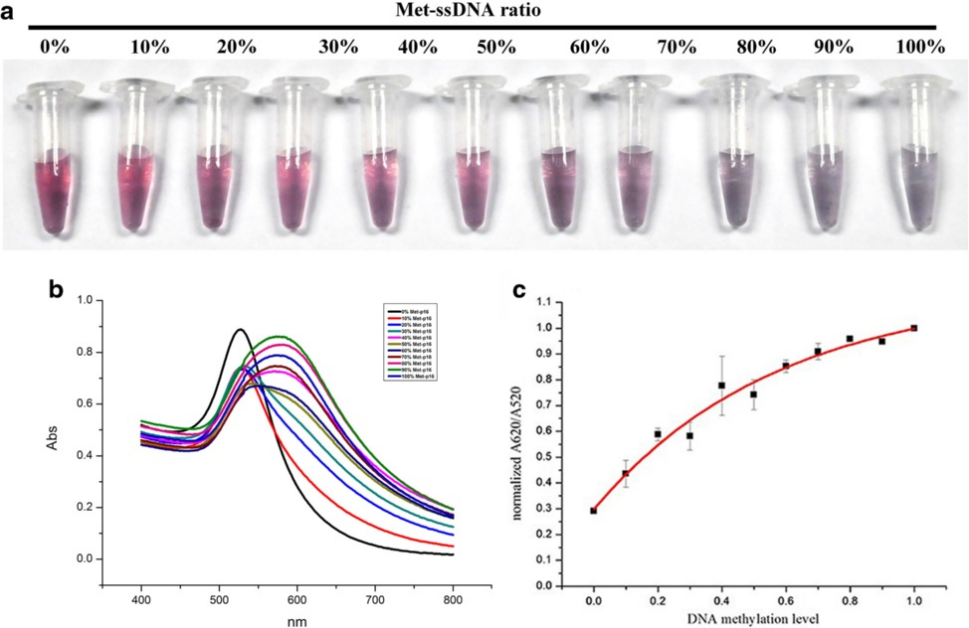
Semi-quantitative detection and analysis of DNA methylation degree in the mimic system. The application of ssDNA-GNP probes for the visual detection of DNA methylation in 5 mol/L NaCl (**a**) and the corresponding UV-vis spectrum (**b**). Curve fitting results of *A*_620 nm_/*A*_520 nm_ value to DNA methylation degree: *Y* = *abX* + *c*, *a* = −0.85, *b* = 0.18, *c* = 1.15, *R* ^2^ = 0.98. *Y* is the *A*_620nm_/*A*_520nm_ value and *X* is the Met-*p16* concentration (**c**)

The probe was incubated with mixtures containing different ratios of Met-*p16* and Dem-*p16*. After annealing, the mixtures were transferred into the 5.0 mol/L NaCl solution. The solution color changed from red to purple and finally to blue when the Met-*p16* ratio increased from 0 to 100 % in the mixture (Fig. 5a). Furthermore, the absorption peak in the UV/vis spectra of GNPs shifted and broadened to a higher wavelength (from 522 to 558 ± 13 nm) with an increasing ratio of Met-*p16* (Fig. 5b). We used *A*_620 nm_/*A*_520 nm_ as the index to indicate the dsDNA-GNP aggregation level [14, 25, 26] and found that there was not a linear correlation between *A*_620 nm_/*A*_520 nm_ values and Met-*p16* quantities (Fig. 5c). Whereas the *A*_620 nm_/*A*_520 nm_ value of a sample with 20 % Met-*p16* was significantly different compared with the control (0 % Met-*p16* sample) (*P* < 0.05), and the results were even more significant when comparing the 40 % Met-*p16* sample and the control (*P* < 0.01, Additional file 1: Figure S4). Statistical analysis indicated that the relationship between DNA methylation quantity and *A*_620 nm_/*A*_520 nm_ values followed the functional equation:

$$ y=1.15-0.85\times {0.18}^x\kern1em {R}^2=0.98 $$

Here, *y* is the normalized to the *A*_620 nm_/*A*_520 nm_ value and *x* is the Met-*p16* ratio.

According to the study by Drexler, the methylation frequency of tumor suppressor gene promoter sites was over 40 % in cancer patients [27]. Jamese et al. investigated aberrant CpG islands in *p16* in 13 colon cancer cell lines and found that the methylation level was as high as 92 % [28]. The detection limit of the constructed probe in the present study was 20 %; thus, the probe has an effective detection range for DNA methylation in HCT116 cells.

### Semi-quantitative Detection of DNA Methylation in Cancer Cell Lines

**Fig 6:**
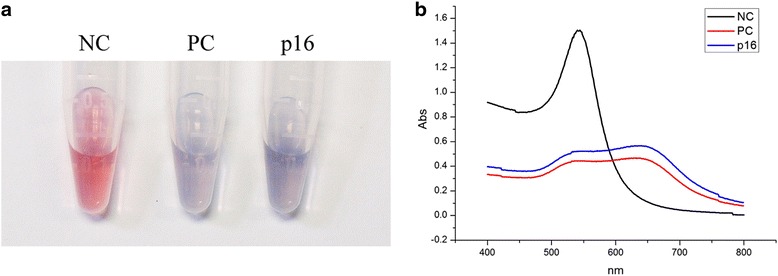
Visual determination of DNA methylation of *p16* in HCT116 cell line. Visual detection of negative and positive controls and the *p16* sample in 5.0 mol/L NaCl solution (**a**). Changes in the UV-spectrum (**b**). *NC* negative control, *PC* positive control, *p16 p16* sample

The targeted sequence (*p16* seq) in the promoter site was achieved through a series of experiments including genomic DNA extraction, bisulfite treatment, PCR amplification (Additional file 1: Figure S5a, c, e, f), endonuclease digestion (Additional file 1: Figure S5b, d, g) and denaturation. Meanwhile, the PC and NC seqs were *Bst*UI digested and then denatured. The mixtures containing *p16* seq were added into the probe solution. Mixtures of PC and NC seq were also treated. After incubating for 5 min at room temperature, the probe solution mixtures were transferred to 5.0 mol/L NaCl solution. The solution color of the *p16* seq group changed from red to violet, as did that in the PC seq group. In the NC seq group, the solution color was red throughout the experiment (Fig. 6a). The UV-vis absorption spectra of the solution showed obvious peak broadening and a red shift from 522 to 641 nm in the *p16* gene seq group. A similar phenomenon was observed for the PC seq; the red shift was from 522 to 633 nm. For the NC seq, there was no UV-vis peak broadening, and the red shift was only 15 nm (Fig. 6b). According to the standard curve (Fig. 5c), *y* = 1.15 − 0.85 × 0.18^*x*^ (y is the normalized *A*_620 nm_/*A*_520 nm_ value, *x* is the Met-*p16* ratio), the DNA methylation level of the *p16* gene in HCT116 cell was detected and semi-quantitatively measured. In our assay, the *A*_620 nm_/*A*_520 nm_ values were 1.11 and 1.13 in the *p16* seq and PC seq groups, respectively, and the normalized *A*_620 nm_/*A*_520 nm_ of *p16* seq was 0.98. The calculated DNA methylation in the *p16* promoter site was 91 % in HCT116 cells.

**Fig 7:**
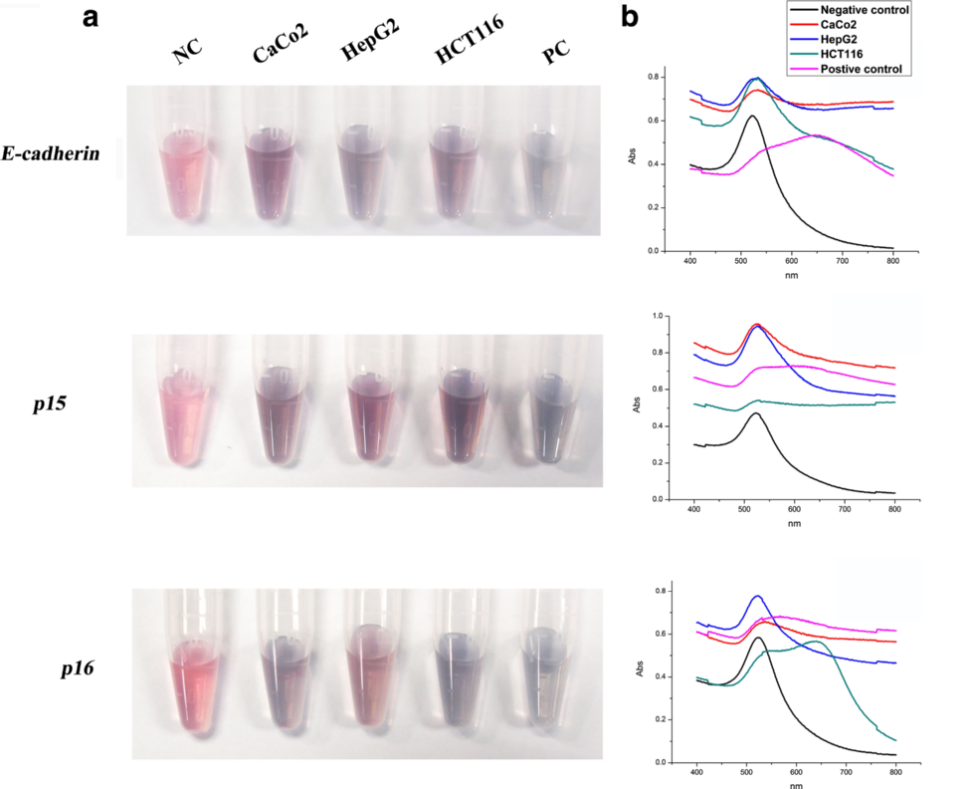
Visual detection of DNA methylation of *E-cadherin*, *p15*, and *p16* in CaCo2, HepG2, and HCT116 cell lines. Visual detection of different samples in 5.0 mol/L NaCl solution (**a**). Changes in UV-spectra (**b**)

As mentioned above, semi-quantitatively assay was broadened to detect the methylation level of *E-cadherin* and *p15* genes in CaCo2, HepG2, and HCT116 cell lines. Meanwhile, DNA methylation of *p16* genes in CaCo2 and HepG2 was also detected. The varied solution color and the corresponding UV-vis absorption spectra were recorded by camera and spectrometer (Fig. 7a, b). According to the normalized *A*_620 nm_/*A*_520 nm_ values, the methylation level was calculated. In the order of CaCo2, HepG2, and HCT116 cell lines, methylation level of *E-cadherin* was 49, 76, and 57 %; *p15* was 52, 34, 79 %; and *p16* was 77, 36, and 91 %. The certain gene in different cancer cell lines was with distinct degree of methylation.

## Conclusions

Based on the SPR characteristics of GNPs, we constructed a GNP probe modified with thiolated ssDNA. The stability and sensitivity of the monodispersed probe was assayed in a mimic condition, and its standard curve was obtained (Fig. 4c, *y* = 1.15 − 0.85 × 0.18^*x*^, *y* is the normalized *A*_620 nm_/*A*_520 nm_ value, *x* is the Met-*p16* ratio). We used the probe to detect and semi-quantify DNA methylation at the promoter site in three tumor suppressor genes (*E-cadherin*, *p15*, and *p16*) in CaCo2, HepG2, and HCT116 cancer cell lines. The result showed that the ssDNA-GNP probes were with the capacity to semi-quantitatively assay methylation levels of tumor suppressor gene in cancer cell lines.

The probe had several advantages. It provides a simple and rapid method for detecting DNA methylation, it has high sensitivity to simultaneously detect methylation in multiple target genes, the reaction endpoint is visually detectable, and DNA methylation can be measured with the corresponding standard curve. We expect that GNP probes might be applied as a novel choice for the early diagnosis of DNA methylation-related diseases.

## Additional file

Additional file 1:
**Figure S1.** Characterization of ssDNA-GNPs constructed at 50 °C. The agarose gel images of ssDNAGNP samples synthesized at 37 °C and 50 °C (a). The SEM images of ssDNA-GNPs synthesized at 50 °C (b). **Figure S2.** (a) The stability of ssDNA-GNPs probes with different lengths of regulatory domains in 0.1 M NaClsolution. (b) The UV-Vis spectra of three specimens. **Figure S3.** After incubated with Dem-p16 (a) and Met-p16 (c), aggregation of the probe 2 in different concentration NaCl solution at room temperature. (b) and (d) are UV-Vis spectrum corresponding to the treated group of Dem-p16 and Met-p16. **Figure S4.** The relationship of Met-p16 ratio and A620nm/A520nm, error bars represent mean ± SD (n=3),*p <0.05, **p <0.01. **Figure S5.** (a,c,e) Bisulfite PCR amplification of E-cadherin, p15 and p16 sequences in CaCo2, HepG2 and HCT116 cell, (f) Nest PCR amplification, (b,d,g) endonuclease digestion with BstUI.
