# Supplementary material for: Utilizing Gold Nanoparticle Probes to Visually Detect DNA Methylation
Source: Nanoscale Res Lett. 2016 Jun 21;11:304. doi: 10.1186/s11671-016-1487-5 (PMC4916073; doi:10.1186/s11671-016-1487-5)
Supplement: Additional file 1: — Figure S1. Characterization of ssDNA-GNPs constructed at 50 °C. The agarose gel images of ssDNAGNP samples synthesized at 37 °C and 50 °C (a). The SEM images of ssDNA-GNPs synthesized at 50 °C (b). Figure S2. (a) The stability of ssDNA-GNPs probes with different lengths of regulatory domains in 0.1 M NaClsolution. (b) The UV-Vis spectra of three specimens. Figure S3. After incubated with Dem-p16 (a) and Met-p16 (c), aggregation of the probe 2 in different concentration NaCl solution at room temperature. (b) and (d) are UV-Vis spectrum corresponding to the treated group of Dem-p16 and Met-p16. Figure S4. The relationship of Met-p16 ratio and A620nm/A520nm, error bars represent mean ± SD (n=3),*p <0.05, **p <0.01. Figure S5. (a,c,e) Bisulfite PCR amplification of E-cadherin, p15 and p16 sequences in CaCo2, HepG2 and HCT116 cell, (f) Nest PCR amplification, (b,d,g) endonuclease digestion with BstUI. [file 11671_2016_1487_MOESM1_ESM.pdf]

Supplementary

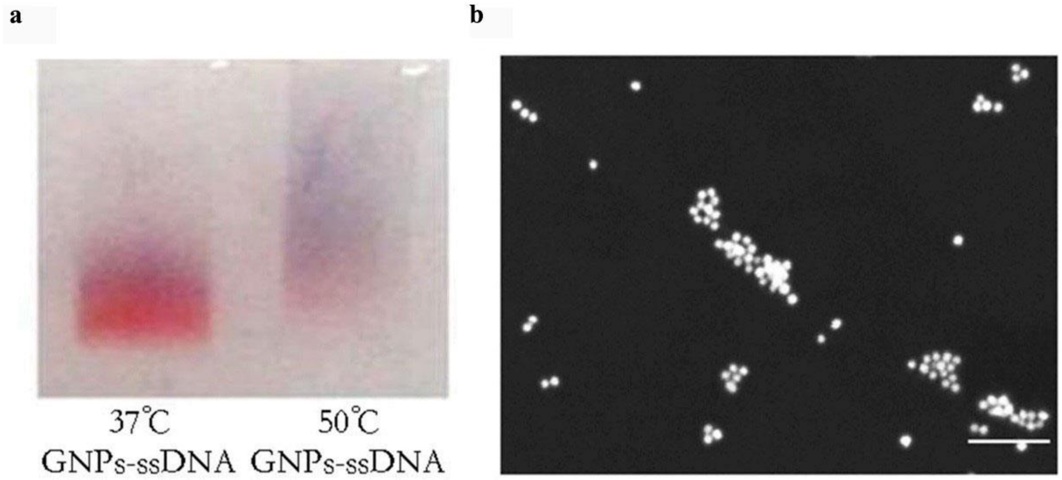

Fig s1 Characterization of ssDNA-GNPs constructed at 50 ℃. The agarose gel images of ssDNA-GNP samples synthesized at 37 ℃ and 50 ℃ (a). The SEM images of ssDNA-GNPs synthesized at 50 ^{o}C (b).

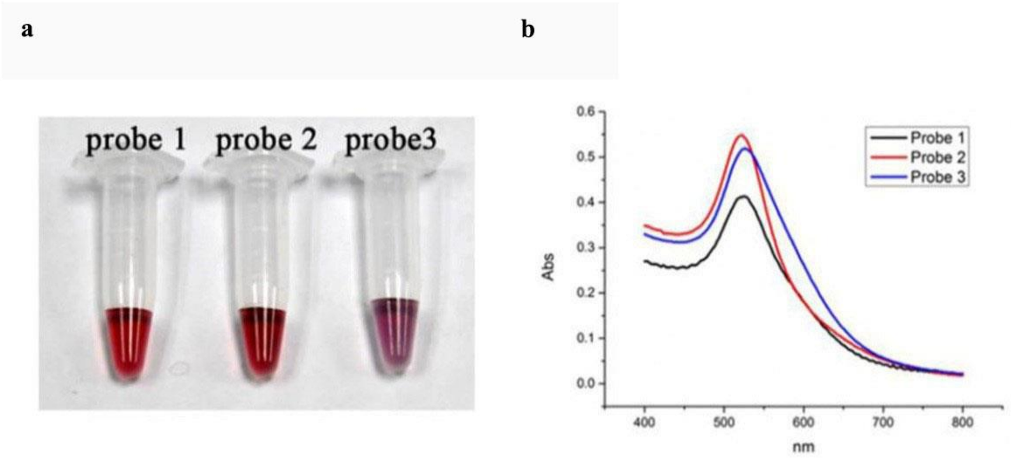

Fig s2 (a) The stability of ssDNA-GNPs probes with different lengths of regulatory domains in 0.1 M NaCl solution. (b)The UV-Vis spectra of three specimens.

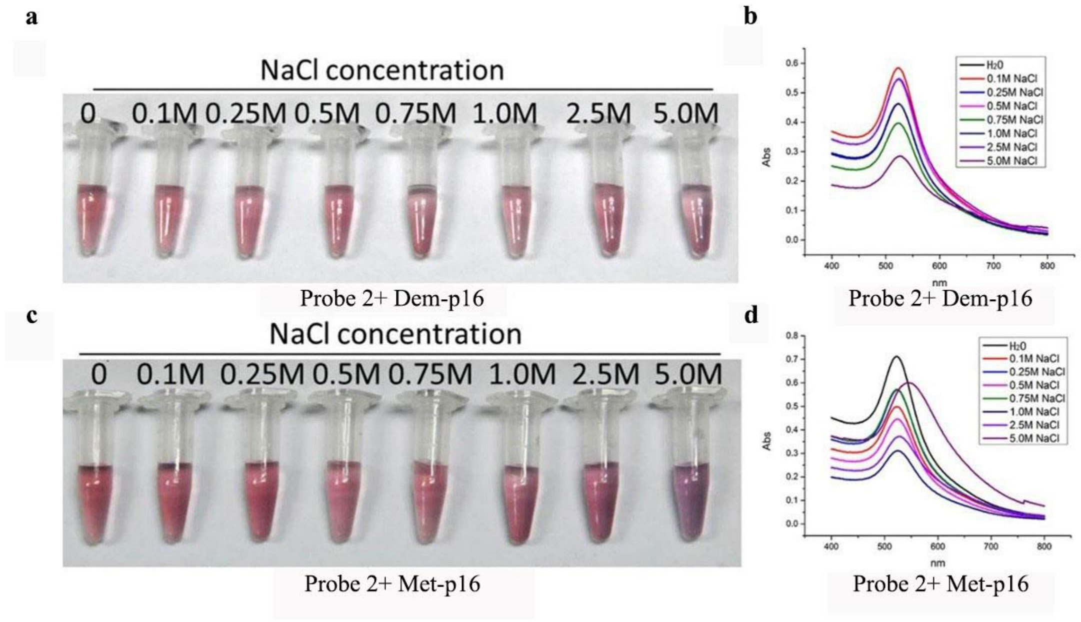

Fig. s3. After incubated with Dem-p16 (a) and Met-p16 (c), aggregation of the probe 2 in different concentration NaCl solution at room temperature. (b) and (d) are UV-Vis spectrum corresponding to the treated group of Dem-p16 and Met-p16.

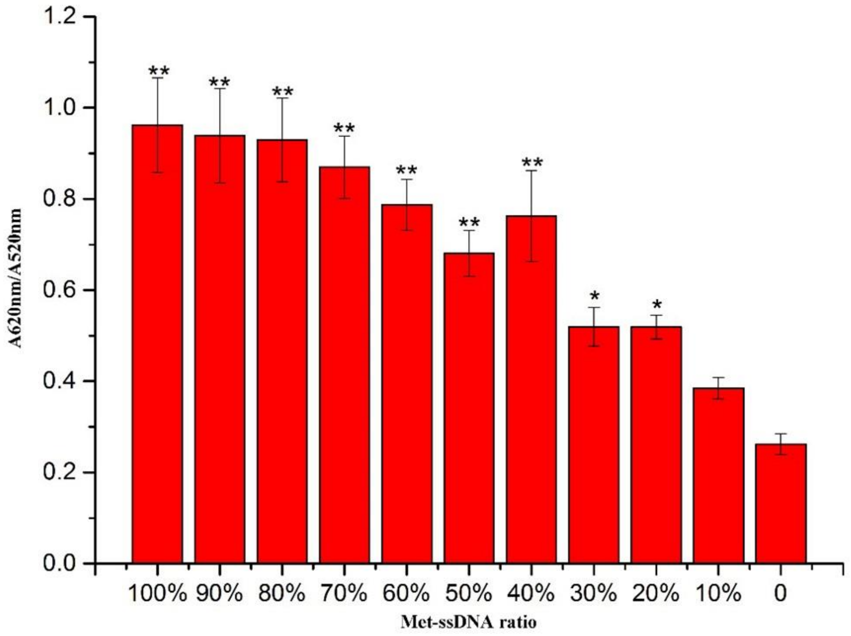

Fig s4 The relationship of Met-p16 ratio and A_{620nm}/A_{520nm}, error bars represent mean ± SD (n=3),*p<0.05, **p<0.01.

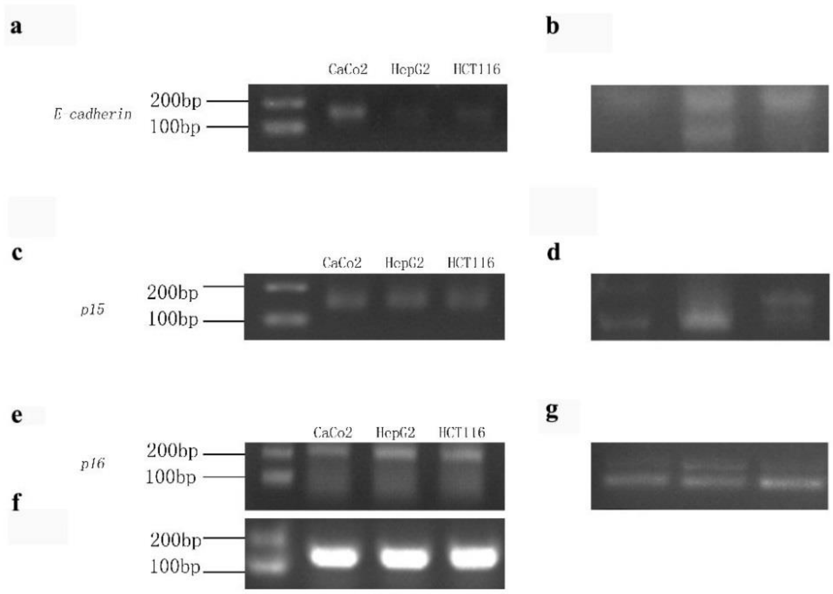

Fig s5 (a,c,e) Bisulfite PCR amplification of *E-cadherin*, *p15* and *p16* sequences in CaCo2, HepG2 and HCT116 cell, (f) Nest PCR amplification, (b,d,g) endonuclease digestion with *Bst*UI.
